# Evaluation of a portable hemoglobin photometer in pregnant women in a high altitude area: a pilot study

**DOI:** 10.1186/1471-2458-9-228

**Published:** 2009-07-11

**Authors:** Xiaoyan Zhou, Hong Yan, Yuan Xing, Shaonong Dang, Bianba Zhuoma, Duolao Wang

**Affiliations:** 1 Department of Epidemiology and Health Statistics, Xi'an Jiaotong University College of Medicine, Xi'an, PR China; 2 Obstetrics and Gynecology Department, Lhasa People's Hospital, Lhasa, PR China; 3 Department of Epidemiology and Population Health, London School of Hygiene and Tropical Medicine, London, WC1E 7HT, UK

## Abstract

**Background:**

Anemia is a widespread public health problem associated with an increased risk of morbidity and mortality, especially in pregnant women. This study examined the agreement between a portable hemoglobin photometer and a laboratory analyzer in determining hemoglobin level in pregnant women.

**Methods:**

This study recruited 69 pregnant women in Tibet, China. Capillary blood samples were taken to measure hemoglobin concentration using the hemoglobin photometer and the laboratory analyzer. Limit of agreement, concordance and intraclass correlation coefficient were used to evaluate the agreement. Laboratory measurement was considered as the standard reference method. Sensitivity and specificity were calculated to assess the error in screening for anemia.

**Results:**

Mean difference between the two methods was -2.1 g/l. wide 95% limits of agreement were found (-22.6 g/l to 18.4 g/l). The intraclass correlation coefficient was 0.795, and concordance correlation coefficient was 0.793. Sensitivity and specificity were 94.9% and 76.7% respectively. Positive predictive value was 84.1%, and negative predictive value was 92.0%.

**Conclusion:**

This hemoglobin photometer is not recommended for determining hemoglobin concentration in pregnancy in a high altitude area.

## Background

Anemia is a widespread public health problem associated with an increased risk of morbidity and mortality, especially in pregnant women [1]. The global prevalence of anemia in pregnant women and non-pregnant women is 41.8% and 30.2% respectively [2]. Anemia during pregnancy is a well-established risk factor for both the mother and the fetus [3]. Hemoglobin assessments are relatively precise and are used to screen individuals for anemia, to assess the iron status of populations, and to evaluate responses to nutritional interventions. The portable hemoglobin meter (HemoCue) has been widely used in recent years [4].

The unreliability of methods used to determine hemoglobin will widen the distribution of hemoglobin values, and may bias the estimates of anemia and its health consequences. Previous evaluations on the performance of the HemoCue device have revealed conflicting results. von Schienck et al. [5] and Morris et al. [6] found a high accuracy with this device compared with standard laboratory methods. However, Chen [7], Rippmann [8] and Neville [9] did not recommend this device in general practice.

Only a few well-documented validation studies have been reported in pregnant women. Hudson-Thomas et al. [10] conducted a study in pregnant women in Jamaica; however, limited information was reported. Only one validation study has been conducted at an altitude of about 1500 meters above sea level in Mexico [11]. However, there is no information on the performance of the HemoCue device when used at a higher altitude. Therefore we conducted a study in pregnant women in Lhasa, Tibet, to assess the agreement of this device with a laboratory hematology analyzer to determine hemoglobin concentration. We also evaluated the performance of this device in anemia screening in this population.

## Methods

### Subjects and study setting

The Tibet Autonomous Region is located in southwest China, and is often called the "Roof of the World" or the "Third Pole". In Lhasa, the capital of Tibet, the average altitude is about 3700 meters above sea level. Natural conditions in this area are extremely harsh. With the rise in elevation, the air pressure and oxygen content per cubic meter of air is reduced, the atmospheric pressure and oxygen content are approximately two-thirds of those at lower levels. This study was conducted in a prefecture level general hospital. Subjects were from the outpatient unit of the Obstetrics and Gynecology department. Pregnant women who came for antenatal examinations consented to take part in the study whereas the pregnant women with serious diseases were excluded. This study is approved by the Research Ethics Committee of Xi'an Jiaotong University College of Medicine.

### Principle of Measurements

The HemoCue^®^B-Hemoglobin system (HemoCue AB, Ängelholm, Sweden) consists of disposable microcuvettes containing reagent in a dry form and a single purpose-designed photometer. The reaction in the microcuvette is a modified azide-methemoglobin reaction. Sodium deoxycholate haemolyses erythrocytes and hemoglobin is released. Sodium nitrite converts hemoglobin to methemoglobin which, together with sodium azide, gives azide-methemoglobin. The absorbance is measured at two wavelengths (570 nm and 880 nm) in order to compensate for turbidity in the sample. The Sysmex KX-21TM (Sysmex Corporation, Kobe, Japan) is an automated blood cell counter intended for in vitro diagnostic use in clinical laboratories. It is a compact, fully automated hematology analyzer with simultaneous analysis of 18 parameters in whole blood mode and capillary blood mode. It measures the hemoglobin concentration using a non-cyanide hemoglobin method (STROMATOLYSERWH). The instrument has been proved to provide accurate and reliable results including hemoglobin concentrations [12,13]. A quality control of the Sysmex KX-21 was achieved before each day of experiments. The instrument measured the control material and compared the results with a normal range of value given by the manufacturer for eight parameters. If the results are outside this range, the apparatus needs to be calibrated.

The calibration procedure was achieved just before start of this study and every month thereafter according to the user guide. At least five samples of a healthy subject are used. Hemoglobin concentrate (HGB) and hematocrit (HCT) of the samples were determined using another calibrated apparatus for 3 times (norm DIN 58931 for HGB and norm DIN 58933 for HCT). The blood samples are then measured with the Sysmex KX-21. A new calibration value was calculated and replaced the old one.

### Data collection

A specially trained nurse interviewed the subjects and executed the HemoCue Measurement. Firstly subjects were asked to sign a consent form and complete a short questionnaire. Then capillary hemoglobin measurement was performed using the HemoCue device according to the manufacturer's instructions. Briefly, a lancet punctured the middle finger of the right hand and a capillary blood sample was taken, the third drop of blood was used to fill the cuvette. When the cuvette was full, it was correctly placed in the HemoCue device immediately. The nurse was trained to conduct the procedure of sampling and measurement. The HemoCue devices used were checked with the device-specific, control cuvette every day. If the value obtained deviated from the assigned value on the control cuvette card more than 3 g/l, this device will be replaced by another calibrated one. The measurement was conducted in an office and a thermometer was used to confirm the proper temperature (15–40 centigrade). Shortly after this, another nurse led subjects to the hospital laboratory where a laboratory hematology measurement of each subject's capillary blood was carried out using a standard procedure. Capillary blood mode is used. Fingertip puncture were made and blood sample were attracted to a capillary pipe. Quality control was run on the analyzers on a daily basis. The nurse was responsible for collect the report of the laboratory measurement.

### Statistical Analysis

Agreement between the HemoCue device and the laboratory analyzer was assessed by the method of Bland and Altman [14], where the mean difference and the standard deviation of the differences between the HemoCue and the laboratory hematology analyzer, and the limits of agreement were calculated. Figures plotting difference against the average were graphed as Bland and Altman recommended [14]. The laboratory measurement was considered the standard in this study, and linear regression analysis was used to calculate the coefficient of determination and to estimate the error in the HemoCue measurements. We used intraclass correlation coefficient (ICC) and concordance correlation coefficient (CCC) to measure the agreement between the two methods. The ICC measures the amount of overall data variance due to between-subject variability, while the CCC is based on the distance in the plane of each pair of data to the 45° line through the origin [15]. We also calculated the prevalence of anemia using the criteria of National Centers for Disease Control and Prevention of the United States (CDC) and CDC altitude adjustment [16,17]. The cut-off point of CDC anemia criteria for pregnancy is 110 g/l in both the first and third trimester and 105 g/l in the second trimester. According to the CDC formula, adjusted hemoglobin concentration at an altitude of 3700 meter will be 28.5 g/l lower than the actual one. The sensitivity, specificity, positive predictive value, negative predictive value and kappa value were calculated to assess the performance of the two methods in screening for anemia.

Data were entered into a Microsoft ACCESS database and analysis was carried out using Stata 8.0 for Windows.

## Results

This study was carried out between October 2006 and October 2007, on 69 pregnant women. Five subjects refused to participate in the project. Table 1 shows the characteristics of the study participants.

**Table 1:** Summary statistics of characteristics of the 69 pregnant women

	Mean	Minimum	Maximum	SD
Age (year)	27.12	19	40	4.84
Gestational age (week)	33.70	5	42	9.50
HCHB^1 ^(g/l)	126.35	55	163	18.02
LAHB^2 ^(g/l)	128.45	78	162	14.84

HCHB^1^, the haemoglobin concentration measured by HemoCue^®^; LAHB^2^, the haemoglobin concentration measured by laboratory analyzer; SD, standard deviation.

**Figure 1:**
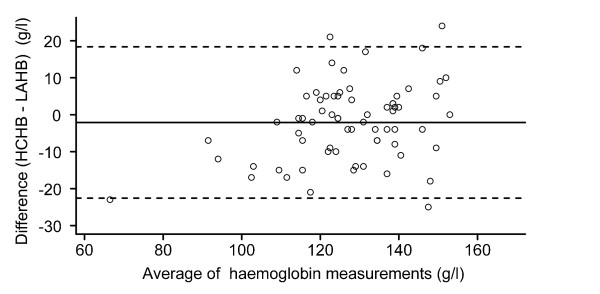
**Difference versus average of Hb measured by HemoCue and the hematology analyzer**. Horizontal line represents mean difference; broken lines represent 95% limits of agreement. Hb, hemoglobin concentration; HCHB, the hemoglobin concentration measured by HemoCue^®^; LAHB, the hemoglobin concentration measured by laboratory analyzer.

**Figure 2:**
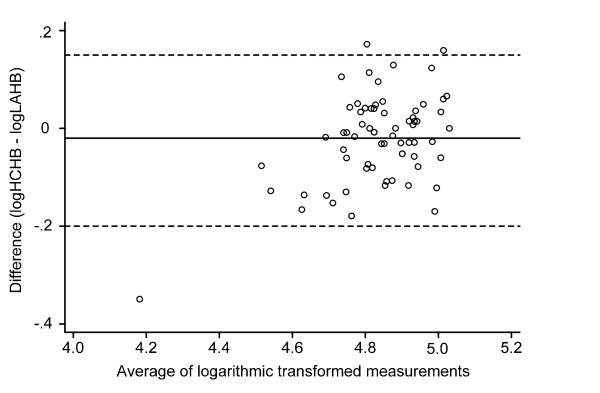
**Difference versus average of Hb measured by HemoCue and the hematology analyzer after logarithmic transformation**. Horizontal line represents mean difference; broken lines represent 95% limits of agreement. Hb, hemoglobin concentration; HCHB, the hemoglobin concentration measured by HemoCue^®^; LAHB, the hemoglobin concentration measured by laboratory analyzer.

The overall mean difference between the two measurements was -2.1 g/l, differing significantly from zero with wide 95% LOA. Figure 1 represents the LOA and individual differences. The 95% LOA were -22.6 g/l and 18.4 g/l, thus the HemoCue may be 22.6 g/l below or 18.4 g/l above the measurement using the laboratory haematology analyzer. The 95% confidence intervals of the LOA were 15.86 g/l to 20.87 g/l, and -25.08 g/l to -20.06 g/l. To assess the trend in the relationship between the difference and the mean, analyses of logarithmic transformed data were carried out. As Figure 2 shows, the difference was then -0.02 and the LOA were -0.20 and 0.15 on a logarithm scale. The LOA then had to be related to the original scale of measurement. When the antilog of these limits were taken we obtained 0.82 and 1.16. These limits tell us that for about 95% of cases, the HemoCue measurement of hemoglobin will be between 0.82 and 1.16 times that of the laboratory measurement. Thus, the HemoCue measurement may differ from the laboratory measurement by 18% below and 16% above. Fifteen (21.7%) of the absolute values of the difference were above 10.0 g/l. Five (7.2%) of the absolute values of the difference were above 20.0 g/l. Fifty-one (73.9%) of the values measured by the HemoCue were within 10% of the corresponding values measured by the laboratory haematology analyzer. Only thirty-six (53.6%) of the values measured by the HemoCue were within 5% of the laboratory measurements.

The Pearson correlation coefficient between the HemoCue hemoglobin concentration and the laboratory hemoglobin concentration was 0.82, and the coefficient of determination was 0.66. The ICC was 0.795, with a 95% confidence interval of 0.690 to 0.868. The CCC was 0.793 (95% confidence interval: 0.710–0.977). These results give a moderate agreement between the two measurements.

When hemoglobin concentrations were altitude-adjusted by the CDC method [17], the overall prevalence of anemia was 68.3% using the HemoCue device and was 56.5% using the laboratory analyzer. The kappa value was 0.729, indicating a moderate agreement. The sensitivity and specificity were 94.9% and 76.7%, respectively, the positive predictive value was 84.1%, and the negative predictive value was 92%, considering the laboratory analyzer as the reference method.

## Discussion

Anemia affects 40% of pregnant women worldwide [2], and is detrimental to the health of mother and their fetus. Hemoglobin concentration is used for detecting anemia and also evaluating the gravity of this disorder. The use of a laboratory hematology analyzer is problematic in remote areas. Portable hemoglobin meters offer a rapid, handy and inexpensive measurement of hemoglobin. In addition, the finger puncture to allow capillary blood sampling as an easy technique is less resource-intensive than vein puncture, and is more acceptable to patients and the community. The present study evaluated the agreement between the HemoCue device and a laboratory hematology analyzer for capillary hemoglobin measurement. This is the first validation study conducted in a high altitude area, thus filling a gap in this area.

The 95% LOA in present study is much wider compared to a study (-2.93 g/l and 3.38 g/l) carried out in infants in Honduras [6] and a study (-3.7 g/l and 4.5 g/l) carried out in patients undergoing aortic surgery [18]. However, several studies have demonstrated a similar LOA to that obtained in this study. Bhaskaram et al. demonstrated a LOA of -33 g/l and 9 g/l in India [19]. Rippmann et al. found a LOA of -6.0 g/l and 18.0 g/l in surgical blood samples [8], while a LOA of -18.0 g/l and 16.8 g/l in a study by van de Louw et al. was obtained in patients gastrointestinal bleeding in France [20].

In our study 21.7% of the absolute values of the difference were above 10.0 g/l. These results are slightly poorer than those reported in previous studies. Radtke et al. reported a difference of greater than 10.0 g/l in 9% of samples from unselected German blood donors [21], and in a study by Rippmann et al. [8], and the figure is 17.9%. van de Louw et al. found 21% of the difference was greater than 10.0 g/l [20]. The results in our study and from the previous studies highlight the need for a pilot validity study before the HemoCue device can be used in a particular population or setting such as high altitude regions. The determination of hemoglobin in capillary blood using the HemoCue device was not accurate in our study, which was similar to validation studies in patients and blood donors. The result consists with a study conducted in Mexico middle high altitude area [11]. We therefore do not recommend this device to measure capillary hemoglobin in pregnancy in high altitude regions due to the discrepancies associated with this device compared to the laboratory hematology analyzer.

ICC and CCC are commonly used to evaluate agreement between measurements. In our study the ICC was 0.795, and the CCC was 0.793. These findings were similar or slightly poorer than those reported in previous studies in general settings [6,11,22]. These findings together with the LOA, confirm that capillary hemoglobin determination using the HemoCue does not have an acceptable agreement with capillary hemoglobin determination using the laboratory hematology analyzer.

In a study in Indonesian mothers, Sari et al. reported a sensitivity of 70.6% and a specificity of 97.5% in detecting anemia [23]. Radtke et al. reported a sensitivity of 98.0% and a specificity of 50.0% in screening blood donors [21], and Sawant et al. found a sensitivity of 99.0% and a specificity of 45.0% in screening blood donors [22]. In another study in Mexico, Neufeld et al. reported a sensitivity of 79% in detecting anemia in adults and 84% in children, and a specificity of 97% and 93% [11], respectively. In the present study, setting the laboratory analyzer as reference method, the sensitivity and specificity for anemia screening were 94.9% and 76.7%, the positive predictive value was 84.1% and the negative predictive value was 92.0%. These results are similar to or slightly better than previous studies, and the results seem acceptable.

Several authors have pointed out that capillary blood samples are more variable than venous blood samples [6,7], which may partly explain the poor agreement observed in our study. A shortcoming of this study is that this study did not measure with both methods using the same puncture. Disagreement may also be affected by the quantity of capillary blood samples, although the only one nurse who conducted the sampling and measurement process had undergone strict trainings.

The sample size in our study meets the general recommendations of Altman of at least 50 subjects in a methods comparison study [24].

This study is the first to evaluate HemoCue device in the determination of capillary blood hemoglobin in pregnant women in a very high altitude area. We also obtained detailed information on the agreement between the portable HemoCue and a laboratory analyzer on hemoglobin levels in pregnancy. Due to poor acceptance among participants, we were unable to obtain duplicate measurements for both methods to assess the reliability of these methods. However, the laboratory analyzer has been reported to perform very well in other published articles. Although we obtained poor agreement between HemoCue device and the laboratory analyzer for the determination of capillary blood hemoglobin, we do not know whether the high altitude in the study area contributed to this poor agreement. This is a topic that needs investigation.

## Conclusion

Capillary hemoglobin determination using the HemoCue does not have an acceptable agreement with capillary hemoglobin determination using the laboratory hematology analyzer. We therefore do not recommend this device to measure capillary hemoglobin in pregnancy in high altitude regions.

## Competing interests

The authors declare that they have no competing interests.

## Authors' contributions

XZ carried out the study and data analyses and drafted the manuscript. HY conceived the study and participated in study design and helped to draft manuscript. YX, SD and BZ participated in study design, coordination and made modifications of the paper. DW participated in the statistical analysis and helped to draft manuscript. All authors read and approved the final manuscript.

## Pre-publication history

The pre-publication history for this paper can be accessed here:


